# Cold unfolding of heat-responsive TRPV3

**DOI:** 10.21203/rs.3.rs-4285061/v1

**Published:** 2024-04-24

**Authors:** Guangyu Wang

**Affiliations:** University of California Davis

## Abstract

The homotetrameric thermosensitive transient receptor potential vanilloid 1–4 (TRPV1–4) channels in sensory neurons are strongly responsive to heat stimuli. However, their cold activations have not been reported in line with the nonzero heat capacity difference during heat or cold unfolding transitions. Here, along with the experimental examinations of the predicted ring size changes in different domains against the central pore during channel gating at various temperatures, the K169A mutant of reduced human TRPV3 was first found to be activated and inactivated by cold below 42°C. Further thermoring analyses revealed distinct heat and cold unfolding pathways, which resulted in different protein thermostabilities. Thus, both cold and heat unfolding transitions of thermosensitive TRPV1–4 channels may exist once a mutation destabilizes the closed state.

## Introduction

Cold and heat unfolding transitions with the relatively low and high melting temperatures (T_m_, _low_ and T_m, high_, respectively) are universal characteristics of proteins if the heat capacity difference (DC_p_) is nonzero during the process. However, cold unfolding often cannot be directly detected once it occurs at a temperature below the freezing point of water. For this reason, globular proteins were experimentally observed in only limited cases of cold unfolding by using nuclear magnetic resonance (NMR) and circular dichroism (CD) and differential scanning calorimetry (DSC). These proteins include metmyoglobin (1), staphylococcus nuclease (2), Streptomyces subtilisin inhibitor (3–4), ß-Lactoglobulin1 (5), barstar (6), bacterial cold shock protein (7), thioredoxin (8), L9 mutants (9–10), yeast Yfh1 and frataxin (11–14), HIV-1 protease monomer (15), *E. coli* IscU (16), and thermophilic multidomain initiation factor 2 (17). However, cold unfolding has never been reported for any membrane protein.

On the other hand, the relatively low melting temperature (T_m, low_) can be available for detecting cold unfolding once *ad hoc* mutations or denaturants such as urea or extreme pH values destabilize globular proteins (1–17). Thereafter, it is fitting to ask whether such a mutation could also increase the T_m, low_ of the heat-responsive thermosensitive transient receptor potential (TRP) vanilloid 1–4 (TRPV1–4) channels for capturing cold unfolding (18). Following the finding of a higher DC_p_ of 8.68 kcal/mol-K along with the high structural and functional thermosensitivities (Ω_10_ and Q_10_=21, respectively) during a heat-evoked channel gating transition from a reduced closed state to an oxidized open state of TRPV3 above 50 °C (19), the K169A mutant has become such a potential candidate because it can be spontaneously open with an increase in an open probability (P_o_) from 0.57 at room temperature to 0.69 at 4 °C along with an inactivated state (20). Furthermore, when a cysteine residue is introduced to the homotetrameric Cys-free K169A construct for fluorescence labeling and förster resonance energy transfer (FRET) assay, the relative fluorescence intensities recorded at A131C in the ankyrin repeat domain (ARD) and L513C in the S2–S3 linker increase upon the stimulation of an agonist carvacrol. However, the relative fluorescence intensities measured at L513C in the S2–S3 linker, A619C in the pore turret and A721C in the C-terminal linker domain decrease from 25 °C to 42 °C in a reversible manner (21). Given that the cryogenic electron microscopy (cryo-EM) 3D structures of wild-type (WT) hTRPV3 and the K169A mutant have been available at 4 °C in different gating states (20), it is necessary to examine such a hypothesis that cold unfolding-induced channel opening and inactivation of the homotetrameric K169A mutant increase the the relative fluorescence intensities determined in the S2–S3 linker, the pore turret and the C-terminal linker domain from 42 °C to 25 °C.

To test this hypothesis, when four identical residues in those three specific domains of mouse or human TRPV3 (mTRPV3 or hTRPV3, respectively) or the K169A mutant formed several rings against the central pore in different redox states, their size differences were compared between closed and open or inactivated states. The resultant changes in the relative fluorescence intensities during channel gating were also predicted and compared with the experimental data at 25 °C and 42 °C (21). In addition, the recently-developed and examined graph theory-based grid thermodynamic model was also used to evaluate two defined thermodynamic parameters of hTRPV3 and the K169A mutant to keep temperature-dependent gating states. First, when noncovalent interactions identified in the high-resolution 3D structure of protein can form a systematic fluidic grid-like mesh network along the single polypeptide chain, the melting temperature threshold (T_m_) for heat unfolding can be determined by the grid size and the strength of grid size-dependent noncovalent interactions in the biggest grid; Second, the systematic thermal instability (T_i_) can be computed as the ratio of the total grid sizes (S) to the total noncovalent interactions (N) along the same single polypeptide chain (19, 22–26). Since both parameters and predicted changes in the relative fluorescence intensities in three specific domains from 42 °C to 25 °C were consistent with the experimental observations, the above hypothesis was confirmed. Further, protein thermostability and detailed comparisons between cold and heat unfolding transitions and the underlying mechanisms are also discussed.

## Results

### Predicted changes in the FRET of oxidized mTRPV3 upon channel opening at 42°C

**Figure 1:**
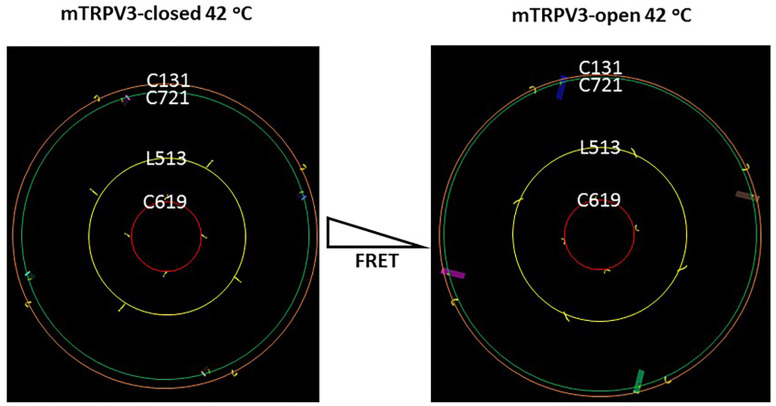
Relative ring size changes in different domains of the oxidized mTRPV3 channel along with channel opening. The homo-tetrameric cryo-EM structures of the oxidized mTRPV3 channel in the closed and open states at 42 °C (PDB ID, 7MIN and 7MIO, respectively) were used for the model. C131, C721, L513 and C619 are located in the ARD, the C-terminal linker domain, the S2-S3 linker and the pore turret, respectively.

In the presence of C612-C619 disulfide bond, four C619 residues in the pore turrets of the homotetramic mTRPV3 formed a ring with the same size during channel opening at 42°C (Fig. 1). However, rings shaped by four residues at C131 in the ankyrin repeat domains, L513 in the S2-S3 linkers and C721 in the C-terminal linker domains significantly increased theirs sizes to a different extent along with channel opening at 42°C (Fig. 1). Therefore, when a fluorescence dye pair, sulfo-cyanine 3 maleimide (Cy3) and sulfo-cyanine 5 maleimide (Cy5), were randomly labeled into cysteines at these positions of four subunits, the FRET intensity would decrease upon channel opening at 42°C. In other words, the relative fluorescence intensities at these three positions would increase along with channel opening.

### Predicted changes in the FRET of reduced hTRPV3 upon the mutation-induced opening at 4°C

**Figure 2:**
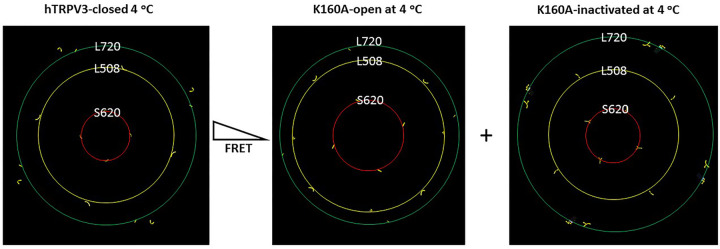
Relative ring size changes in different domains of the reduced hTRPV3 channel during channel gating. The homo-tetrameric cryo-EM structures of the reduced hTRPV3 channel in the closed state at 4 °C (PDB ID, 6UW4), the K169A mutant channels in the open state at 4 °C (PDB ID, 6UW6) and in the inactivated state at 4 °C (PDB ID, 6OT2) were used for the model. L720, L508 and S620 are located in the C-terminal linker domain, the S2-S3 linker and the pore turret, respectively.

In the absence of C612-C619 disulfide bond, the rings lined by four residues at L508 in the S2-S3 linkers and S620 in the pore turrets of the homotetramic hTRPV3 became significantly larger in the open state of the K169A mutant than in the closed state of the WT construct at 4°C (Fig. 2). Accordingly, if the K169A mutant shared the similar structure with the WT hTRPV3 in the closed state along the PC-dependent minimal gating pathway from D396 in the pre-S1 domain to K705 in the TRP domain, once Cy3 and Cy5 were randomly attached to cysteines at these positions of four subunits, the FRET intensity of the K169A mutant would also decrease upon channel opening at 4°C. In other words, the relative fluorescence intensities at these two positions would also increase along with channel opening.

Notably, four L720 residues in the C-terminal linker domains of the K169A mutant produced a larger ring in the inactivated state than in the open state (Fig. 2). Thus, the predicted relative fluorescence intensities in the C-terminal linker domain would increase along with channel inactivation (Fig. 2).

Taken together, given that the measured relative fluorescence intensities at these three critical positions in the C-terminal linker domains, the S2-S3 linker, and the pore turret decrease from 25 to 42°C (21), the K169A mutant may inactivate along with channel opening from 42°C to 25°C if its closed state is structurally similar to that of the WT construct (20). In this case, the relevant thermoring analyses are necessary to examine if the WT hTRPV3 could stably maintain closed above 42°C, and if the K169A mutant could stably keep open or inactivated up to 25°C. However, owing to a low resolution of inactivated K169A in the detergents (27), only the high-resolution closed and open states in MSP2N2 could be further analyzed to establish the melting temperature thresholds (T_m_) for heat unfolding and the relevant systematic thermal instability (T_i_).

### Reduced hTRPV3 can be closed below 51°C

**Figure 3:**
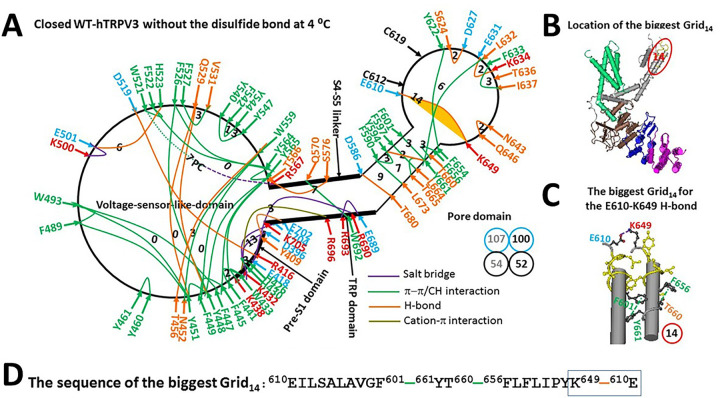
The grid-like noncovalently interacting mesh network along the PC-dependent minimal gating pathway of the reduced hTRPV3 channel in a closed state at 4 °C. (A) The topological grids in the systemic fluidic grid-like mesh network. The cryo-EM structure of one subunit of the reduced and closed hTRPV3 channel in MSP2N2 at 4 °C (PDB ID, 6UW4) was used for the model. The pore domain, the S4-S5 linker, the TRP domain, the VSLD and the pre-S1 domain are indicated in black. Salt bridges, p interactions, and H-bonds between paired amino acid side chains along the PC-dependent minimal gating pathway from D396 to K705 are marked in purple, green, and orange, respectively. The grid sizes required to control the relevant noncovalent interactions were computed with graph theory and are labeled in black numbers. The E610-K649 H-bond in the biggest Grid_14_ is highlighted. The dashed line is the putative PC bridge between W521 and R567. The total grid sizes and grid size-controlled noncovalent interactions along the PC-dependent minimal gating pathway are shown in the cyan and black circles, respectively. The grey and black numbers were calculated in the presence and absence of the putative PC bridge, respectively. (B) The location of the biggest Grid_14_. **(C)** The structure of the biggest Grid_14_ with a 14-residue size to control the E610-K649 H-bond in the pore domain. **(D)** The sequence of the biggest Grid_14_ to control the E610-K649 H-bond in the blue box.

Reduced hTRPV3 was structurally similar to reduced mTRPV3 at 4°C. However, it had a weaker lipid density at the active vanilloid site (20). Assuming the same phosphatidylcholine (PC) lipid was bound to W521 and R567 via a π interaction and a salt bridge (Fig. 3A) (19), the total number of noncovalent interactions and the total grid sizes along the defined PC-dependent minimal gating pathway from D396 in the pre-S1 domain to K705 in the TRP domain were 54 and 107, respectively. Thus, the systematic thermal instability (T_i_) was calculated as 1.98 (Table 1). When the PC lipid was released, Ti was about 1.92 as a result of a decrease in the total noncovalent interactions and the total grid sizes from 54 and 107 to 52 and 100, respectively. Therefore, the PC lipid at the vanilloid site had a weak effect on the thermal stability of closed and reduced hTRPV3.

Notably, when compared with the reduced form of the closed mTRPV3 channel, although the R416-D519 H-bond of the reduced hTRPV3 channel was observed along with the T411-D519 H-bond broken at the interface between the pre-S1 domain and the volatge sensor like domain (VSLD), the presence of the new E418-R690 salt bridge at the pre-S1/TRP interafce and the new Q570-W692 π interaction at the S4-S5 linker/TRP interface produced a smaller thermoring to control the R416-D519 H-bond. It had a 3-residue size via the shortest path from R416 to E418, R690, E689, R693, W692, Q570, T566, Y565, Y564, F522, W521, D519 and back to R416 (Fig. 3A).

On the other hand, there was the biggest Grid_14_ in the pore domain (Fig. 3A). It had the shortest path from E610 to F601, Y661, T660, F656, K649, and back to E610 to control the E610-K649 H-bond/salt bridge between two pore turrets (Fig. 3B–D). With 2.5 equivalent basic H-bonds sealing this biggest Grid_14_, the calculated T_m_ for heat unfolding was at least 51°C (Table 1), which was approximately the measured threshold of 50°C for the heat-evoked channel opening of the reduced hTRPV3 channel (30). In further agreement with this notion, the relative fluorescence intensities measured at A721C in the C-terminal linker domains of the WT hTRPV3 construct are the same at 25°C and 42°C (21). Hence, if the reduced K169A mutant shared the same thermoring structure with with WT hTRPV3 along the PC-dependent minimal gating pathway from D396 to K705 in the closed state, it could maintain closed from 4°C to 51°C.

### Reduced K169A can be open up to 32°C

**Figure 4:**
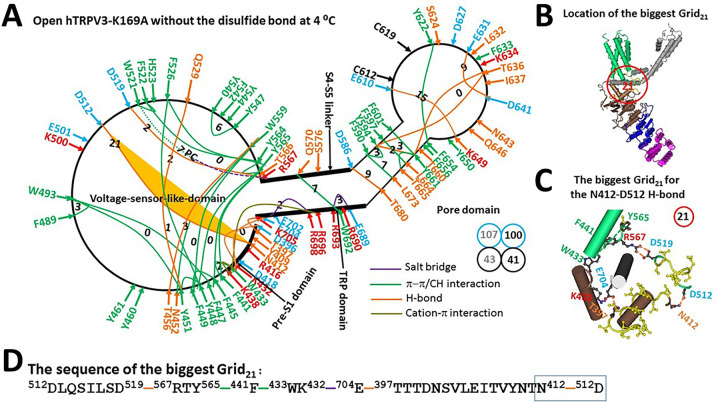
The grid-like noncovalently interacting mesh network along the PC-dependent minimal gating pathway of the reduced hTRPV3-K169A channel in a open state at 4 °C. **(A)** The topological grids in the systemic fluidic grid-like mesh network. The cryo-EM structure of one subunit of the reduced and open hTRPV3-K169A channel in MSP2N2 at 4 °C (PDB ID, 6UW6) was used for the model. The pore domain, the S4-S5 linker, the TRP domain, the VSLD and the pre-S1 domain are indicated in black. Salt bridges, p interactions, and H-bonds between paired amino acid side chains along the PC-dependent minimal gating pathway from D396 to K705 are marked in purple, green, and orange, respectively. The grid sizes required to control the relevant noncovalent interactions were computed with graph theory and are labeled in black numbers. The N412-D512 H-bond in the biggest Grid_21_ is highlighted. The dashed line is the putative PC bridge between W521 and R567. The total grid sizes and grid size-controlled noncovalent interactions along the PC-dependent minimal gating pathway are shown in the cyan and black circles, respectively. The grey and black numbers were calculated in the presence and absence of the putative PC bridge, respectively. **(B)** The location of the biggest Grid_21_. **(C)** The structure of the biggest Grid_21_ with a 21-residue size to control the N412-D512 H-bond at the pre-S1/VSLD interface. **(D)** The sequence of the biggest Grid_21_ to control the N412-D512 H-bond in the blue box.

When the K169A mutation disrupted the swapping K169-E751 salt bridge (20), the R416-D519 H-bond at the pre-S1/VSLD interface, together with the E418-R690 salt bridge at the pre-S1/TRP interface, was also broken at 4°C (Fig. 4A). As a result, When the Y448-Q529 and E501-H523 H-bonds were disconnected, the Y409-E702 H-bond was replaced with the T397-E704 one at the pre-S1/TRP interface, and the T566-S576 H-bond was substituted by the D519-R567 one at the S4-S5 linker/VSLD interface. In that case, even if the E610-K649 H-bond in the biggest Grid_14_ of the closed state was intact along with the PC lipid at the vanilloid site, the channel was still open along with a new biggest Grid_21_ at the VSLD/pre-S1/TRP interfaces (Fig. 4B–C). It had a 21-residue size via the shortest path from D512 to D519, R567, Y565, F441, W433, K432, E704, T397, N412 and back to D512 to control the D512-N412 H-bond at the pre-S/VSLD interface (Fig. 4D). As 1.5 equivalent basic H-bonds sealed this biggest Grid_21_, the melting temperature threshold (T_m_) for heat unfolding was calculated as 32°C (Table 1). In that regard, the K169A mutant could be still open below 32°C.

It has been reported that the lipid density at the active vanilloid site is enhanced along with the mutation-induced channel opening at 4°C (20). If the same PC lipid was bound to W521 and R567 (19), when the total number of noncovalent interactions along the PC-dependent minimal gating pathway from D396 to K705 decreased from 54 to 43, the total grid sizes factually kept the same value of 107. Thus, the systematic thermal instability (T_i_) increased from 1.98 to 2.49 (Table 1). Even if the PC lipid was released, the T_i_ was still as high as 2.44 when the total number of noncovalent interactions and the total grid sizes were 41 and 100, respectively. In this regard, the open state of the K169A mutant was actually unstable even if a temperature has been lowered down to 4°C.

## Discussion

Cold unfolding of most proteins is difficult to detect when it occurs at temperatures well below water freezing. Therefore, except for Yfh1 and (Apo)-IscU, which may be the limited examples of natural proteins with an observable cold unfolding under (quasi) physiological conditions (31–32), *ad hoc* mutations or denaturants such as urea or extreme pH values are usually used to destabilize globular proteins to increase a relatively lower melting temperature (T_m, low_) in favor of detecting cold unfolding (1–17). Similarly, although cold unfolding of membrane proteins is much more difficult to investigate structurally because of the effects of various membrane environments on protein stability, the K169A mutation could destabilize the closed state in favor of cold unfolding for detailed structural studies (20). Therefore, once cold unfolding-induced channel opening and inactivation of the K169A mutant from a resting closed state were confirmed by the temperature-dependent changes in the relative fluorescence intensities in different domains, this mutant renders an ideal template for possible mechanistic insight into cold unfolding below 42°C when compared with the WT construct, which can be activated by heat unfolding above 50°C.

### Asymmetric cold and heat unfolding transitions

**Figure 5:**
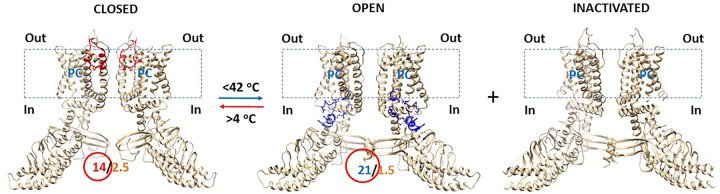
Tentative model for the cold activation and inactivation of reduced hTRPV3-K169A from a closed state. **(A)** The homo-tetrameric cryo-EM structures of hTRPV3 in the resting closed state (PDB ID: 6UW4) and hTRPV3-K169A in the open and inactivated states (PDB ID, 6UW6 and 6OT2, respectively) were used for the model. For convenience, only two opposite subunits are shown for three gating states. The dashed rectangles are the membrane areas. In the presence of the PC lipid (blue) at the active vanilloid site, hTRPV3-K169A has 2.5 equivalent basic H-bonds (orange) to seal the biggest Grid_14_ (red) in the pore domain so that the mutant channel is closed below a calculated threshold of 51 °C. When the temperature decreases below 42 °C, cold unfolding at the protein-water interface activates the mutant channel along with the new biggest Grid_21_ (blue) at the pre-S1/VSLD interface, which is sealed by 1.5 equivalent basic H-bonds (orange). Meanwhile, K169A also inactivates.

Although both heat and cold unfolding transitions decrease the total numbers of noncovalent interactions along the PC-dependent minimal gating pathway from D396 in the pre-S1 domain to K705 in the TRP domain (Table 1) (19), they have the different mechanisms based on the behavior of the Gibbs–Helmholtz equation. Heat unfolding is an endothermic process so that heat can break the K614-N647 H-bond in the biggest Grid_11_ of reduced mTRPV3 or the E610-K649 H-bond in the biggest Grid_14_ of reduced hTRPV3 to initiate channel opening above 50°C (Figs. 3, 5) (19). In contrast, cold unfolding is an exothermic process. Therefore, cold cannot directly disconnect such an enthalpically favorable H-bond in the biggest Grid_11_ or Grid_14_. Instead, when the charged residues at the VSLD/pre-S1/TRP interfaces are exposed to interfacial water, more ordered water molecules may form networks of H-bonds linking with R416 and E418 in the pre-S1 domain at low temperature to compete with D519 in the VSLD and R690 in the TRP domain, respectively (Figs. 3–5). In this case, even if the PC lipid was not released from the active vanilloid site, as a result of the broken R416-D519 and E418-R690 H-bonds, the D519-R567 H-bond could still replace the T566-S576 one, stimulating channel opening in the same way as heat unfolding does (Figs. 3 & 4) (19).

In support of the above proposal, cold unfolding of yeast frataxin has been reported when the electrostatic hinge, which is formed by a special quadrilateral arrangement of negative residues, D86-E89-E90-E93 mapped in the β-sheet, allows the entrance of water molecules into the hydrophobic core (12). Furthermore, more water was observed at the hydrophobic ligand binding site in electron density maps of Hsp90α with a ligand bound upon cryo-cooling from room temperature (33). Finally, more protein-water H-bonds were found in the cold-denatured state of yeast frataxin than in the hot-denatured state (34), and the strong electrostatic interactions between ions and water molecules stabilize yeast frataxin (35). Thus, a protein interaction with the surrounding water may play a critical role in the cold unfolding of the reduced hTRPV3-K169A channel. In any way, unfolding by either heat or cold is not complete as a cooperative cyclization reaction against a decyclization reaction also contributes to the thermal stability of such a membrane protein (Figs. 3–5).

### Distinct effects of heat and cold unfolding transitions on the protein thermostability

Generally, the total non-covalent interactions (N) along the lipid-dependent minimal gating pathway may determine the entropy of the open conformations of side chains for unfolding. In contrast, the total grid sizes (S) along the same gating pathway may control the intra-chain interactions and the entropy of compact conformations of main chains for refolding (36). Therefore, the systematic thermal instability (T_i_ = S/N) can be an optimal parameter for evaluating the structural thermostability of proteins.

When the T_i_ of the reduced mTRPV3 channel along the PC-dependent minimal gating pathway from D396 to K705 declines from 1.88 to 1.20 upon heat unfolding above 52°C, the heat-evoked channel open state is much more thermostable than the resting closed state (19). In contrast, the cold-evoked open state of the K169A mutant channel was much more thermally unstable than the resting closed state because the T_i_ raised from 1.98 to 2.49 upon cold unfolding below 42°C (Table 1). In that regard, heat unfolding may induce a more compact and structured open state along the PC-dependent minimal gating pathway (19). However, cold unfolding evoked a more expanded state. These observations were consistent with the notion that the unfolded proteins become more compact when the temperature is raised but disordered at low temperature (37). That may be the reason for the inactivation of the K169A mutant to be accompanied by the cold-evoked channel opening (Figs. 2, 5) (20). In any way, for the homotetrameric TRPV3 channel, the rings formed by four identical residues in the S2-S3 linkers always increase their sizes along with channel pore opening induced by cold or heat unfolding or carvacrol.

## Conclusion

Proteins undergo both cold and heat unfolding transitions, but cold unfolding often cannot be directly detected once the temperature is naturally below the freezing point of water. Here, the cold unfolding of the thermosensitive membrane protein, the reduced K169A mutant of the human TRPV3 channel, was first identified in atomistic detail above the freezing temperature of water after the predicted ring size changes against the central pore in different domains during channel gating were validated experimentally at distinct temperatutres. Therefore, the cold unfolding of a membrane protein can also be detected after the artificial destabilization of a closed state.

## Materials and Methods

### Data mining resources

The cryo-EM 3D structures of the closed and open mTRPV3 channels with the C612-C619 disulfide bond in cNW11 at 42°C were sampled as controls to predict the FRET changes in this study (PDB ID, 7MIN, model resolution = 3.09 Å; 7MIO, model resolution = 3.48 Å, respectively) (28). The cryo-EM 3D structures of the reduced hTRPV3 channel with MSP2N2 in the closed state (PDB ID, 6UW4, model resolution = 3.20 Å) and the reduced hTRPV3-K169A channels with MSP2N2 in the open (PDB ID, 6UW6, model resolution = 3.70 Å) and inactivated (PDB ID, 6OT2, model resolution = 4.1 Å) states were further sampled to track the FRET results and to analyze cold unfolding (20, 27).

### Filtering noncovalent interactions

The stereo-elective or regio-selective inter-domain diagonal and intra-domain lateral noncovalent interactions along the PC-dependent minimal gating pathway of hTRPV3 or the K169A mutant from D396 to K705 were filtered by using UCSF Chimera and the same strict and consistent standard as previously used and confirmed (19, 22–26). They included salt-bridges, lone pair/CH/cation-π interactions and H-bonds between paired amino acid side chains. Details of the specific cutoff distances and interaction angles for the different noncovalent interactions are shown in the online Supporting Information (Table S1-S2).

### Mapping of topological grids by using graph theory

The same protocol as described and validated previously was used to map the systematic fluidic grid-like noncovalent interaction mesh network in this study (19, 22–26). After a grid size for each noncovalent interaction was constrained by using the graph theory and the Floyd–Warshall algorithm (38), the uncommon sizes are marked in black numbers on the network map alongside the Grid_x_ with an x-residue size. Moreover, the total number of noncovalent interactions (N) and the total grid sizes (S) along the PC-dependent minimal gating pathway of hTRPV3 or the K159A mutant from D396 to K705 were computed and are shown in the black and cyan circles, respectively, beside the mesh network map for the calculation of the systematic thermal instability (T_i_ = S/N).

### Calculation of the melting temperature threshold

The melting temperature threshold (T_m_) for the heat unfolding of a given grid was calculated by using the same equation as calibrated by the structural data of several proteins such as class I and II fructose aldolases aldolases and TRPV1 and TRPV3 and TRPM8 (19, 22–26):

(1) $${\text{T}}_{\text{m}}{(}^{{}^{\circ}}\text{C})=34+(\text{n}-2)\times 10+\left(20-{\text{S}}_{\max}\right)\times 2$$

where, n is the total number of basic H-bonds (~ 1 kcal/mol for each) energetically equivalent to the noncovalent interactions controlled by the given grid, and S_max_ is the size of the given grid. Thus, the grid’s heat capacity will increase with a decrease in the grid size or an increase in equivalent basic H-bonds.

## Figures and Tables

**Table 1:** Comparison of heat- and cold-induced thermoring structural changes of TRPV3 along the PC-dependent minimal gating pathway from D396 to K705. The comparative parameters are highlighted in bold.

PDB ID	7MIN	7MIO	6UW4	6UW6
**Construct**	mTRPV3	mTRPV3	hTRPV3	hTRPV3-K169A
**Lipid PC at the active vanilloid site**	bound	free	bound	bound
**Redox state**	oxidized	oxidized	reduced	reduced
**Lipid environment**	cNW11	cNW11	MSP2N2	MSP2N2
**Sampling temperature, °C**	42	42	4	4
**Gating state**	Closed	Open	Closed	Open
**# of the biggest grid**	Grid_17_	Grid_9_	Grid_14_	Grid_21_
**Biggest grid size (S**_**max**_ **)**	17	9	14	21
**# of basic H-bonds to controlled S**_**max**_	2.0	2.5	2.5	1.5
**Total non-covalent interactions**	53	49	54	43
**Total grid sizes, a.a**	64	59	107	107
**Calculated T**_**m**_, **°C**	**40**	**61**	**51**	**32**
**Systemic thermal instability (T**_**i**_ **)**	1.21	1.20	1.98	2.49
**Experimental T**_**m**_, **°C**	**42**	**62**	**50**	**25–42**
**Refs for Experimental T**_**m**_, **°C**	[28]	[29, 30]	[30]	[20, 21]

## Data Availability

All data generated or analysed during this study are included in this published article and Supporting Information.

## Abbreviations

ARD: ankyrin repeat domain
CD: circular dichroism
cryo-EM: cryogenic electron microscopy
DSC: differential scanning calorimetry
FRET: förster resonance energy transfer
DC_p_: heat capacity difference
T_m, high_: high melting temperature
T_m, low_: low melting temperature
NMR: nuclear magnetic resonance
Ω_10_: structural thermosensitivity
Q_10_: functional thermosensitivity
PC: phosphatidylcholine
P_o_: open probability
Cy3: sulfo-cyanine 3 maleimide
Cy5: sulfo-cyanine 5 maleimide
T_i_: systematic thermal instability
T_m_: melting temperature threshold
TRP: transient receptor potential
TRPV: TRP vanilloid
TRPVi: (i=1, 2, 3, 4)
hTRPV3: human TRPV3
mTRPV3: mouse TRPV3
VSLD: voltage-sensor-like domain
WT: wild-type
